# Editorial: Serving vulnerable and marginalized populations in social and educational contexts

**DOI:** 10.3389/fpsyg.2023.1310260

**Published:** 2023-10-27

**Authors:** Anies Al-Hroub, Sally Reis, Joseph Madaus, Itab Shuayb

**Affiliations:** ^1^Department of Education, American University of Beirut, Beirut, Lebanon; ^2^Neag School of Education, University of Connecticut, Storrs, CT, United States; ^3^Neag School of Education, University of Cambridge, Cambridge, United Kingdom

**Keywords:** vulnerable adolescent/youth, marginalized adolescents, refugees and asylum seekers, gifted and talented students, twice exceptional learner

In this editorial, we offer a comprehensive overview of the themes and findings from the Research Topic, emphasizing the significance of addressing the needs of vulnerable and marginalized populations in social and educational contexts. We also explore the research included in the Research Topic, which delves into the unique challenges faced by individuals from diverse backgrounds. This interdisciplinary Research Topic provides evidence-based studies on these groups, focusing on various areas such as education, psychology, emotional wellbeing, social work, health, and welfare issues. We believe that this Research Topic of 26 published articles serves as a valuable resource for cutting-edge research and policy development for a wide range of professionals, including scholars, policymakers, psychologists, educators, sociologists, advocates, and practitioners. We are especially proud that the research and policy articles span diverse cultural, ethnic, and linguistic backgrounds in both developing and developed countries.

## Educational accommodations and inclusion

The first theme explored in this Research Topic revolved around educational accommodations and the pursuit of inclusion, as students with disabilities often encounter numerous hurdles in accessing quality education (Shuayb, 2020). A study by Lovett delved into two equity-related concerns regarding educational accommodations, underscoring the necessity of inclusive practices within educational institutions. It was evident that creating inclusive environments required proactive measures to ensure equitable access to educational resources. Another study by Madaus et al. explored the experiences of postsecondary students with disabilities during the COVID-19 pandemic and the subsequent academic year, revealing significant challenges related to accessibility, instruction, and mental health support. In a study by Gelbar et al., the perceptions of accessibility service providers regarding college students with autism spectrum disorder (ASD) were explored, highlighting the critical role of self-advocacy, independent decision-making, self-regulation, and coping strategies in facilitating the success of college students with ASD. These skills empowered students to navigate the academic environment effectively and advocate for their needs. Additionally, a study by Mohamed and Elhoweris focused on preschool teachers' perceptions of gifted learners in Abu Dhabi, emphasizing the need for educational systems to invest in resources and professional development to cater to the diverse learning needs of all students, including those with exceptional abilities. In summary, these studies collectively underscored the pressing need for equitable educational accommodations and support services. They also highlighted the persistence of accessibility challenges, the importance of skill development for students with disabilities, and the significance of early identification and support for gifted learners.

## Impact of COVID-19 on vulnerable populations

The global COVID-19 pandemic had far-reaching consequences, disproportionately affecting vulnerable and marginalized populations (Kantamneni, 2020). In a study conducted by Goodrich et al., the impact of the pandemic on elementary school teachers' practices and perceptions was examined across two critical semesters. The study highlighted the challenges faced by teachers as they adapted to rapidly changing circumstances. In another study, Mitwalli et al. investigated the double burden of COVID-19 and Israeli military rule on persons with disabilities in the West Bank of the occupied Palestinian territory. Their findings illuminated the importance of understanding the unique challenges faced by these populations during times of crisis. A separate study, conducted by Shuayb and Doueiry, focused on the impact of the COVID-19 pandemic on the education and healthcare services of persons with disabilities in Lebanon. In this study, the negative effects of the COVID-19 pandemic were found to bring new challenges to the lives of persons with disabilities. The study identified five overarching themes that hindered persons with disabilities during the COVID-19 pandemic, including accessing education and healthcare, both at the policy and decision levels and in academic and healthcare services.

## Gifted education and talent development

Equity in education also extends to gifted and high-potential students and their talent development (Al-Hroub, 2023; Jouni and Al-Hroub, 2023). A study conducted by Smedsrud et al. explored the experiences of mathematically gifted students. The study underscored the significance of recognizing and nurturing the talents of gifted individuals, regardless of their backgrounds. Furthermore, another study by Chagas-Ferreira et al. delved into the development of talent among the Sinti and Calon Romani populations, providing insights into the importance of recognizing and supporting giftedness within marginalized communities. “*The School Handbook for Dual and Multiple Exceptionality*,” authored by Yates and Boddison in 2020 and reviewed by Batrouni and Al-Hroub, was the subject of this book review. This book review highlighted the significance of the book's themes and chapters in advancing our understanding and approach to addressing dual and multiple exceptionalities in educational settings. This set of articles underscored the profound and disproportionate impact of the global COVID-19 pandemic on vulnerable and marginalized populations, including elementary school teachers, persons with disabilities in the West Bank, and individuals with disabilities in Lebanon. These findings emphasized the urgent need for comprehensive support and policy measures to address the challenges faced by these populations during times of crisis, as well as a focus on strengths as well as deficits (Reis et al., 2022).

## Refugees and marginalized populations

The impact of conflict and displacement on vulnerable populations, particularly children and adolescents, cannot be understated (Al-Hroub et al., 2020). A systematic review of empirical evidence on art therapy with traumatized refugee children and youth, completed by Annous et al., underscored the potential of art therapy as a means of healing and recovery for those who had experienced trauma. Additionally, a study by Khamis explored the role of neuroticism as a mediator and moderator between war atrocities and psychopathology in Syrian refugee children and adolescents. Recognizing the role of individual differences in coping with trauma could inform tailored interventions and support systems for these young survivors. Alkhateeb et al. systematic review study highlighted the often-overlooked challenges faced by parents of children with autism spectrum disorder (ASD) in Arab countries. Understanding the unique stressors and support needs of these families was essential for promoting the wellbeing of both children with ASD and their caregivers. The COVID-19 pandemic disrupted education worldwide, but a study by Brigandi et al. provided a biological systems theory approach to understanding the challenges faced by educators during this crisis. Recognizing the complexities of teaching in a pandemic could inform more effective strategies for remote and hybrid learning, especially for marginalized students. Xiong and Xu shed light on the experiences of Chinese migrant children and their struggles with relative deprivation and loneliness. Their research underscored the importance of addressing the social and emotional wellbeing of marginalized children who faced unique challenges related to migration. The resilience of Palestinian refugees in Shatila camp, Lebanon, was explored in Al Beainy and El Hassan's study. Their research highlighted the coping strategies employed by this marginalized population and their potential for personal growth even in the face of adversity. These studies collectively remind us of the pressing need for compassion, support, and research that amplifies the voices and experiences of marginalized populations, including refugees.

## Transition and mobility in education

Another critical theme explored in this Research Topic was the impact of transition and mobility on educational outcomes. For example, Stamp et al. highlighted the importance of considering seasonal and developmental timing in school mobility and high school dropout rates. The study emphasized the need for comprehensive strategies to support mobile students during critical transitions in their educational journeys. In another study, Ching et al. examined the transition experiences of community college transfer students within an Asian educational context. Their findings shed light on the challenges and opportunities faced by these students as they navigated the “2 + 2” pathway. These studies underscored the significance of timing and support for mobile students during critical transitions, offering insights into the challenges and opportunities they encounter along their educational journeys.

## Social and psychological factors in education

Education is not solely about curriculum; it is also profoundly influenced by social and psychological factors, and Taylor and Zaghi explored the interplay of ADHD characteristics and executive functioning with the GPA and divergent thinking of engineering students. This study underscored the importance of considering individual differences in educational contexts and providing appropriate support. Cody et al. investigated teachers' beliefs about giftedness, with a focus on twice exceptional and culturally, linguistically, and economically diverse populations. This study highlighted the need for culturally sensitive and inclusive approaches to identifying and nurturing gifted students, challenging stereotypes, and recognizing potential in all corners of society. Antoun's study took place in Lebanon, where she explored the framing of education for gifted Lebanese and gifted refugee students. The research provided a critical perspective on the challenges faced by these exceptional students and the importance of fostering their potential in a context marked by adversity and displacement. Jaber and Al-Hroub's qualitative study delved into school counselors' perceptions of virtual counseling in Lebanon. Their findings shed light on the evolving landscape of mental health support in education, particularly in times when virtual platforms play a vital role. Finally, environmental education emerged as a crucial component of responsible citizenship. A study conducted by GAO and Jiang focused on the development and validation of China's Environmental Citizenship Questionnaire among junior high school students. This study highlighted the importance of nurturing environmental awareness and responsibility among future generations. These studies within this thematic area provided educators, policymakers, and researchers with essential insights to navigate the intricate web of social, psychological, and environmental factors that shaped the educational experiences of students in diverse contexts.

## Conclusion

In conclusion, the research presented in this Research Topic underscores the critical importance of serving vulnerable and marginalized populations in social and educational contexts. These populations face unique challenges, and addressing their needs requires a concerted effort from educators, institutions, and policymakers. As we navigate the ever-changing landscape of education, it is imperative that we continue to advocate for equitable and inclusive practices that empower all individuals to reach their full potential. The studies in this Research Topic provide valuable insights and serve as a catalyst for continued research, policy development, and advocacy in support of vulnerable and marginalized populations in education. It is our collective responsibility to ensure that no one is left behind in the pursuit of knowledge and opportunity.

## Author contributions

AA-H: Conceptualization, Writing—original draft, Writing—review & editing. SR: Writing—review & editing. JM: Writing—review & editing. IS: Writing—review & editing.
